# Supplementary material for: Chromothripsis during telomere crisis is independent of NHEJ, and consistent with a replicative origin
Source: Genome Res. 2019 May;29(5):737–49. doi: 10.1101/gr.240705.118 (PMC6499312; doi:10.1101/gr.240705.118)
Supplement: Supplemental Material [file supp_gr.240705.118_Supplemental_file_1.zip › contigs/annotated_contigs/DB82/contig.2.DB82_length_406_mean_cov_6.28078817734.docx]

**DB82_length_406_mean_cov_6.28078817734**

```
GTAGAATTTTGATTTATGAAATAGTACAACAGAGCAAAAGGTATTACTCACATCTGTACTAGATGAGATATTTATAACACAACTGCTAT
 >chr6:19525211-19525487 + E=9e-155
CAGCTCCCAAATTTCAAAACCTTTTAAACCACTTTAGCTTAGAGTACATTTCAAACACATTTTCAACTAGAGATCAACATTCAAAAGTA

CTTTAAAAAATACAAGTTAGAGAAAATATTCAAACTCTTTAGTATAAATATAGCATCTTAGTATCTCTAATAATAATTGGTAGTGTGAG

ATTTTCTCA|C|AGCAGTTCTAACTGGTTCCAAAGATGATCTAGTTCAGAGTTGGCTATCTAACTAAGACGATATGCCAAATCCTAATG
           >chr6:19526053-19526182 + E=4e-66
TTTTCACTCCTTGCTTGAGGAGAGTGTTGTCACTGTGGTTTAAGGAAGATTT
```
